# Deep Compressed Communication and Application in Multi-Robot 2D-Lidar SLAM: An Intelligent Huffman Algorithm

**DOI:** 10.3390/s24103154

**Published:** 2024-05-16

**Authors:** Liang Zhang, Jinghui Deng

**Affiliations:** School of Electrical Engineering and Automation, Anhui University, Hefei 230093, China

**Keywords:** deep compressed network, multi-robot system, Huffman encoder, 2D-lidar SLAM, communication-limited application

## Abstract

Multi-robot Simultaneous Localization and Mapping (SLAM) systems employing 2D lidar scans are effective for exploration and navigation within GNSS-limited environments. However, scalability concerns arise with larger environments and increased robot numbers, as 2D mapping necessitates substantial processor memory and inter-robot communication bandwidth. Thus, data compression prior to transmission becomes imperative. This study investigates the problem of communication-efficient multi-robot SLAM based on 2D maps and introduces an architecture that enables compressed communication, facilitating the transmission of full maps with significantly reduced bandwidth. We propose a framework employing a lightweight feature extraction Convolutional Neural Network (CNN) for a full map, followed by an encoder combining Huffman and Run-Length Encoding (RLE) algorithms to further compress a full map. Subsequently, a lightweight recovery CNN was designed to restore map features. Experimental validation involves applying our compressed communication framework to a two-robot SLAM system. The results demonstrate that our approach reduces communication overhead by 99% while maintaining map quality. This compressed communication strategy effectively addresses bandwidth constraints in multi-robot SLAM scenarios, offering a practical solution for collaborative SLAM applications.

## 1. Introduction

Recent years have witnessed significant advancements in multi-robot SLAM algorithms due to their potential applications in search and rescue operations, environmental monitoring, and the exploration of unknown or hazardous environments [1,2,3,4,5]. Single-robot SLAM, where a solitary robot independently navigates and maps its surroundings, is often constrained by time-consuming exploration and limited observation capabilities in large areas. In contrast, multi-robot SLAM has the advantage of efficiently exploring large-scale environments, enhancing mapping accuracy, and improving robustness through the collaborative efforts of multiple robots in various challenging scenarios.

Traditional multi-robot SLAM is developed directly from expanding the single SLAM into multiple robots under a centralized scheme. For example, the CCM-SLAM [6] stands out as a well-established centralized system for visual-inertial multi-robot SLAM. In this system, a central server takes charge of multi-robot map management, fusion, and optimization. By building upon [6], COVINS [7,8] is an extension that has been demonstrated to scale effectively to 12 robots. Another recent addition to centralized CSLAM systems is CVIDS [9], which has achieved the collaborative localization and dense reconstruction of multiple agents in a unified co-ordinate system by using a monocular visual-inertial sensor suite and a centralized loosely coupled framework. LAMP [10,11] is a computationally efficient and outlier-resilient centralized multi-robot SLAM system that consists of a scalable loop closure detection module and GNC (graduated nonconvexity)-based pose map optimization module. Though centralized systems are simply realized and with high precision, they require stable communications and are not robust to the failure of the central node.

Distributed systems can alleviate these problems by reducing the reliance on a central server. Ref. [12] proposed a metric-semantic 3D mesh model for a distributed multi-robot SLAM system, which includes an outlier-resistant fully distributed trajectory estimation method based on GNC and a novel outlier-resistant distributed PGO algorithm. In [13], Kimera-Multi [12,14] is improved to adapt to large-scale real-world deployments, with special emphasis on handling intermittent and unreliable communications. DOOR SLAM [15] uses a distributed frontend to detect inter-robot loop closures without exchanging raw sensor data, successfully rejecting outliers and obtaining accurate trajectory estimates while requiring low communication bandwidth. At the same time, lidar-based distributed multi-robot SLAM is also underway. In [16], the Scan-Context algorithm is proposed to project a 3D point cloud onto a low-resolution 2D plane, thus converting it into a compact feature vector for retrieving the created map and enabling localization, greatly reducing the amount of data and computational complexity. In [17], a distributed backend is used to remove inter-robot loop closure redundancy and perform position map optimization to optimize the global robot cluster position.

The selection of appropriate map representations of multi-robot SLAM directly impacts its performance on computation efficiency, memory usage, and communication burden. For instance, in environments where ground robots navigate indoor spaces, employing a 2D map is sufficient [18]. Studies have demonstrated that occupancy grid maps provide a compact and accurate solution compared to feature-based maps [19,20]. However, certain applications necessitate 3D representations despite the associated computational and storage complexities. This complexity presents challenges, particularly for resource-constrained robotic platforms. Given the communication limitations in multi-robot SLAM systems, there is a preference for compact or sparse map representations, such as the topological maps used in [21,22]. Additionally, efforts are underway to develop semantic-based representations, such as sparse maps annotated with labeled regions [23]. The choice of map representation is critical for long-term operations due to increasing memory requirements, posing a persistent challenge in multi-robot SLAM [24]. Communication bottlenecks in multi-robot SLAM systems typically arise from the exchange of sensor data or representations used for computing inter-robot loop closures [25]. Robots must exchange sufficient data to determine if other robots have explored the same areas and subsequently estimate map alignment using overlapping map sections. Therefore, advancements in the frontend of multi-robot SLAM systems often involve efficient methods for searching loop closure candidates across a team while considering communication constraints. The increasing challenge of balancing efficientinformation transmission with limited communication bandwidth is a pressing issue in multi-robot SLAM systems.

Distributed multi-robot SLAM highly relies on the exchange of data among the network to realize co-ordination processes, such as place recognition, relative pose computation, loop closure detection, and so on. There is increasing work attempting to relieve the communication burden such that the multi-robot system can be deployed to critical environments with less communication capacity. Instead of communicating the merged full map, only the key points and descriptors from parts of the keyframes (using the camera) or submaps (using lidar) are exchanged to realize these co-ordination processes. As depicted in Table 1, segments are extracted in [26] from the source point cloud to generate a source map that contains lists of low-dimensional segment descriptors. In [9], BRIEF descriptors are extracted from each frame and exchanged with another robot. In [27], the keyframes and map points of each robot are transmitted to the server and then distributed by the server to other robots. In [28], two communication modes are designed in different situations: compact and greedy. In compact mode, the communication overhead is minimized by sending compact descriptors. In greedy mode, each drone shares as much information as possible, which is suitable for good network conditions. In [17], NetVlAD descriptors are transmitted between the robots to perform place recognition. In [12], the data flow between different robots are key points and feature descriptors. Compact representations have been explored, incorporating semantic features [29] that rely on objects as landmarks. This approach requires communicating only object labels and poses to other robots, presenting a condensed object-based descriptor that depends on the configuration of objects in a scene for place recognition. In addition to compacting representations, ensuring the sharing of only pertinent information is valuable. However, the data types transmitted by these methods are partial or compact representations of the data, which will lead to the loss of detail. In a wide range of task scenarios, if there will be an accumulation of errors, it is meaningful to study the transmission of the merged full map.

We present a multi-robot SLAM system utilizing 2D occupancy grid maps as a representation of the environment, enabling merged, full-map transmission. Due to the specific format characteristics of raster maps, we have devised intelligent compression algorithms aimed at significantly reducing redundancy in the information contained within these maps while preserving their overall integrity. These intelligent compression algorithms consist of a lightweight network for both map feature extraction and recovery, along with a map feature coder and decoder.

The main innovations and contributions of this paper are as follows:

1. We present a compressed communication framework for multi-robot SLAM, enabling merged, full-map transmission among robots, which can reduce the duration of exploration and produce a map of the whole environment quickly. Such a whole map plays a key role in many robotic tasks, such as path planning, collision avoidance et al.
2. According to the characteristics of the occupancy grid map, we designed an intelligent compression algorithm by combining a convolutional neural network, Huffman coding, and RLE coding that compress the transmitted full map by both image downsampling and stream encoding.
3. We use Ultra-Wide Band (UWB) as a communication medium to validate the multi-robot SLAM system proposed in this paper. The results show that our method is able to reduce the communication burden by up to 99 percent and the localization error is less than 5 cm compared to when no compression is employed.

The remainder of the paper is organized as follows: In Section 2, we describe the proposed multi-robot SLAM method based on an intelligent compression algorithm. In Section 3, the experimental results are provided to validate the performance of the intelligent compression algorithm. Finally, we conclude this paper in Section 4.

## 2. The Compressed Communication Approach

**Figure 1:**
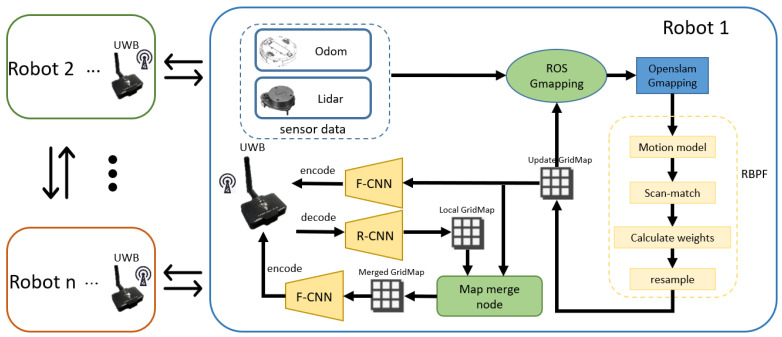
The framework for compressed communication application in multi-robot SLAM.

The multi-robot SLAM framework based on compressed communication consists of the following three main components: full-map creation, full-map compression and transmission, and full-map fusion. In Figure 1, the robots first use Gmapping to build the full map. The map is then compressed by our proposed algorithm and transmitted to other robots using the UWB device. After receiving maps, a robot can decompress maps and merge them with its own created full maps. Eventually, the robot can navigate through the environment using the merged maps.

### 2.1. Full-Map Creation

Gmapping [30] is a SLAM algorithm that combines particle filtering and FastSLAM. The algorithm predicts the robot’s position using a motion model and then uses sensor data for position correction and map updates. As the robot moves through the environment, it gradually builds an accurate occupancy grid map and is able to determine the robot’s position on the map. We use Gmapping to create full maps for each robot.

### 2.2. Full-Map Compression and Decompression

**Figure 2:**
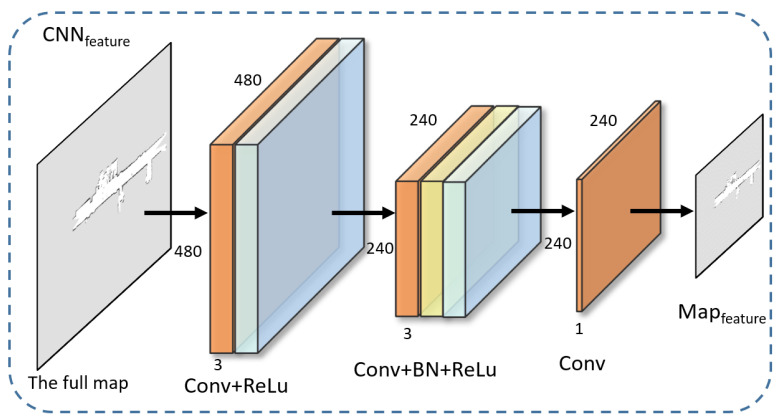
Map feature CNN.

Map compression consists of a map feature extraction CNN ${\mathrm{CNN}}_{feature}$ and an encoder. The map feature $m_{feature}$ that retains the key information of the full map is generated by ${\mathrm{CNN}}_{feature}$. Then, $m_{feature}$ is fed into the encoder to form a string encoding *e*. Corresponding to the above, map decompression consists of a decoder and a map recovery CNN ${\mathrm{CNN}}_{recovery}$. The decoder recovers *e* into $m_{feature}$, and then the $m_{feature}$ is processed through ${\mathrm{CNN}}_{recovery}$ to obtain the new full map. Although the new full map is slightly different from the map before compression, it does not affect its ability to provide navigation for the robot, as demonstrated in the experiments in Section 3.

(1) ${\mathrm{CNN}}_{feature}$: As shown in Figure 2, ${\mathrm{CNN}}_{feature}$ consists of three weight layers, which preserve the structural information of the input map while obtaining storage space-saving $m_{feature}$. The first layer consists of convolution (set the convolution kernel to 3×3) and ReLU (enhance the model’s expressive power) for the purpose of extracting the features of the map. Considering the computing power of the processing unit on the robot, we set 16 sets of 3 × 3 filters. The second layer, composed of convolution, Batch Normalization (BN), and ReLU, aims to downsample and enhance features. In order to change the map resolution to a quarter of the original, the parameter stride of the convolution was set to 2, and 16 filters of size 3×3×16 were used. In the last layer, a filter of size 3×3×16 is used to get the $m_{feature}$. Compared to downsampling the map directly, the $m_{feature}$ obtained by ${\mathrm{CNN}}_{feature}$ contains more map information.

**Figure 3:**
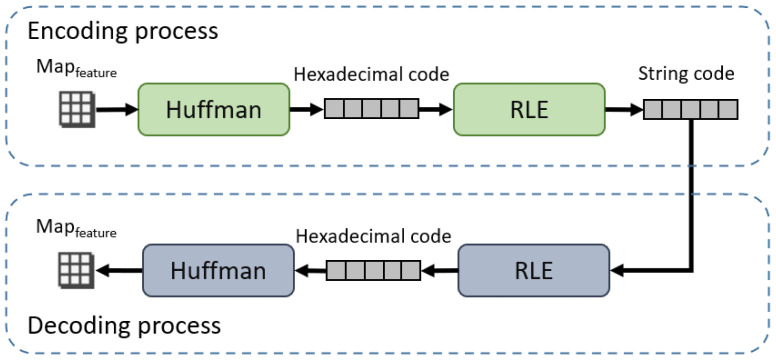
Encoding and decoding process.

**Figure 4:**
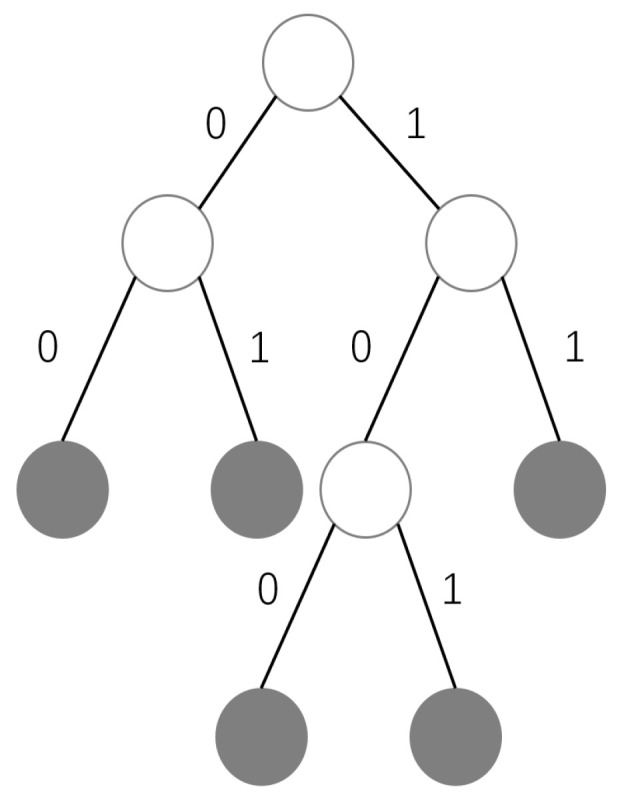
The Huffman coding tree.

**Figure 5:**
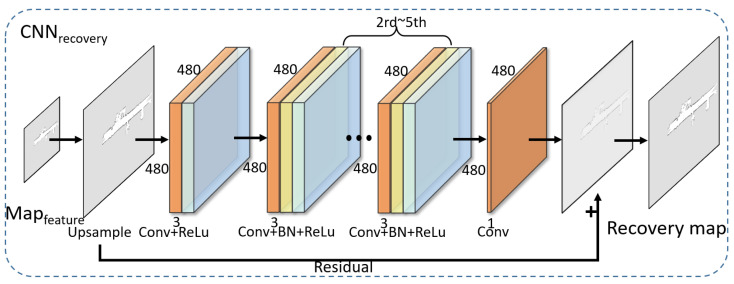
Map recovery CNN.

(2) *Encoder for $map_{feature}$*: The encoding process of $m_{feature}$ is shown in Figure 3. The algorithm of $m_{feature}$ encoder based on RLE and Huffman is shown in Algorithm 1. Firstly, the values that appear in the $m_{feature}$ are recorded $V=\{v_{1},v_{2},...,v_{n}\}$, and the probability of each value in $m_{feature}$ are calculated. We order the probability of occurrence of each value from highest to lowest to obtain the set $P=\{p_{1},p_{2},...,p_{n}\}$, where $p_{i}$ represents the probability of the i-th value in $m_{feature}$. $N=\{n_{1},n_{2},...,n_{n}\}$, which are initialized according to $P=\{p_{1},p_{2},...,p_{n}\}$, and $n_{i}$ corresponds to $p_{i}$. Then, the two smallest values in the set N are merged into a new node, so N and P are updated to $\left\{p_{1},...,p_{n-2},p_{merged}\right\}$, and $\left\{n_{1},...,n_{n-2},n_{merged}\right\}$. We repeat this step until only one node remains in N. According to the process of node emergence, we can get the Huffman coding tree, e.g., Figure 4, where the grey nodes represent the initial nodes and the white nodes are the new nodes generated by merging the initial nodes. As a result, we can encode each value in $m_{feature}$ to obtain the code table $T=\{{\mathrm{code}}_{1}:v_{1},...,{\mathrm{code}}_{n}:v_{n}\}$. Then, according to the code table T, $m_{feature}$ is encoded into the binary code. Finally, the binary code is converted into hexadecimal code h to compress the length of the code. In order to further enhance compression efficiency and reduce communication pressure, consecutive repeated characters in hexadecimal encoding are subsequently processed. We replace the consecutively repeated code segments in h with the repeated characters themselves and the number of times they are repeated to obtain e. Therefore, $m_{feature}$ is compressed into a code table T and a string e.
**Algorithm 1** $m_{feature}$ compression algorithm**Input:** $m_{feature}$   1:record the values in the $m_{feature}$$V=\{v_{1},v_{2},...,v_{n}\}$, count the probability of different value $P=\{p_{1},p_{2},...,p_{n}\}$, initialize nodes $N=\{n_{1},n_{2},...,n_{n}\}$   2:**repeat**   3:   merge the two nodes with the lowest probability to get the new node and update P,N   4:**until** there is only one node left in the set N   5:encode each value according to the Huffman encoding tree to get code table $T:\{{\mathrm{code}}_{1}:v_{1},...,{\mathrm{code}}_{n}:v_{n}\}$   6:rewrite $m_{feature}$ data into binary code according to the code table   7:convert binary code to hexadecimal code h   8:Initialize e as an empty string and count as 1.   9:For index from 1 to length(h) − 1:
    a.Set currentChar as the character at the current index.    b.Set previousChar as the character at the previous index.    c.If currentChar equals previousChar, count + 1.    d.If currentChar does not equal previousChar, append count and previousChar to e, then reset count to 1.
**Output:**
 T, e

(3) *Decoder for $m_{feature}$*: The decoding process of $m_{feature}$ is shown in Figure 3. The algorithm of the decoder is shown in Algorithm 2. First, the e obtained by the compression algorithm is restored to hexadecimal code d. Then, the hexadecimal code d is converted into the binary code. According to the code table T, we can replace the corresponding code with the corresponding value in $m_{feature}$. Finally, $m_{feature}$ is recovered according to T and e.
**Algorithm 2** $m_{feature}$ decompression algorithm**Input:**
 T, e   1:Initialize d as an empty string.   2:While there are characters remaining in e:
    a.Extract the next count-value pair from e.    b.Append count occurrences of the associated character to d.
   3:convert hexadecimal code d to binary code   4:Recover $m_{feature}$ from binary code according to the code table T**Output:**
 $m_{feature}$

(4) ${\mathrm{CNN}}_{recovery}$: In Figure 5, ${\mathrm{CNN}}_{recovery}$ consists of six weighting layers, and there are three types, namely Convolution + ReLU, Convolution + BN + ReLU, and Convolution. The purpose of ${\mathrm{CNN}}_{recovery}$ is to recover the full map from $m_{feature}$, and a residual block is employed in ${\mathrm{CNN}}_{recovery}$. The jump connections of the residual block can help the network learn the difference between the input and the output. This allows ${\mathrm{CNN}}_{recovery}$ to better recover the full map, thus preserving the detailed information of $m_{feature}$. A total of 16 filters of size 3×3 are used to generate 16 feature mappings with ReLU in the first layer. For the second to fifth layers, 16 filters of size 3×3×16 are utilized, and BN is inserted between Convolution and ReLU. The last layer uses a filter of size 3×3×16 to generate the final single-channel output, i.e., the full map.

(5) *Learning algorithm*: The entire network consists of ${\mathrm{CNN}}_{feature}$, the encoder, the decoder, and ${\mathrm{CNN}}_{recovery}$. The optimization function for the network is designed as follows:

(1) $$\left(\bar{\theta_{1}},\bar{\theta_{2}}\right)=\arg\min_{\theta_{1},\theta_{2}}{∥R\left(\theta_{2},Cod\left(F\left(\theta_{1},m\right)\right)\right)-m∥}^{2}$$

where R(·) and F(·) represent ${\mathrm{CNN}}_{recovery}$ and ${\mathrm{CNN}}_{feature}$, respectively. Cod(·) represents the encoder and decoder. *m* is the full map that is the input of the network. $\theta_{1}$ and $\theta_{2}$ are the parameters of the optimization function.

Therefore, we calculate the mean square error between the map of network output and the original full map as the loss and the gradient of the whole network parameters concerning loss by backpropagation. Then, we use the Adaptive Moment Estimation (Adam) optimization algorithm to update the parameters $\theta_{1}$ and $\theta_{2}$ to minimize the optimization function.

(6) *Code transmission based on UWB:* When $m_{feature}$ is processed by the encoder, it becomes a string. In order to facilitate the transmission, we split the string and transmitted it to other robots using UWB segment by segment. After a string has been transmitted, the integrity of the received string is judged. If any data are missing, the receiver commands the transmitter to resend the string.

### 2.3. Multi-Occupancy Grid Maps Fusion

We consider two cases of multi-occupancy grid map fusion:

(1) The initial positions of the robots are known: In this case, the rigid transformation (R,t) between the maps can be computed directly from the initial position of the robot (the x,y co-ordinates and the yaw angle) so that accurate fusion results can be obtained.

(2) $$R=\left[\begin{array}{cc} cos\theta & -sin\theta \\ sin\theta & cos\theta \end{array}\right],t=\left[\begin{array}{c} t_{x}\\ t_{y} \end{array}\right]$$

The (R,t) of the two maps are known; then, the two maps are fused as follows:

(3) $$map_{fusion}=map_{1}+\left(map_{2}\times R+t\right)$$

(2) The initial positions of the robots are unknown: We used the approach in [31] to estimate the transformation relationship between maps using feature matching when the initial position is unknown. This requires that the maps have enough overlapping regions for reliable matching. The approach detects the features in each map, computes a match for the map pair, estimates the transform using RANSAC, and computes a confidence score for each match. Then, the matches with sufficient confidence are selected for map fusion. In this way, fusion can be performed in the case of overlapping maps, even if the initial positions of the robots are not known.

## 3. Experiments

**Figure 6:**
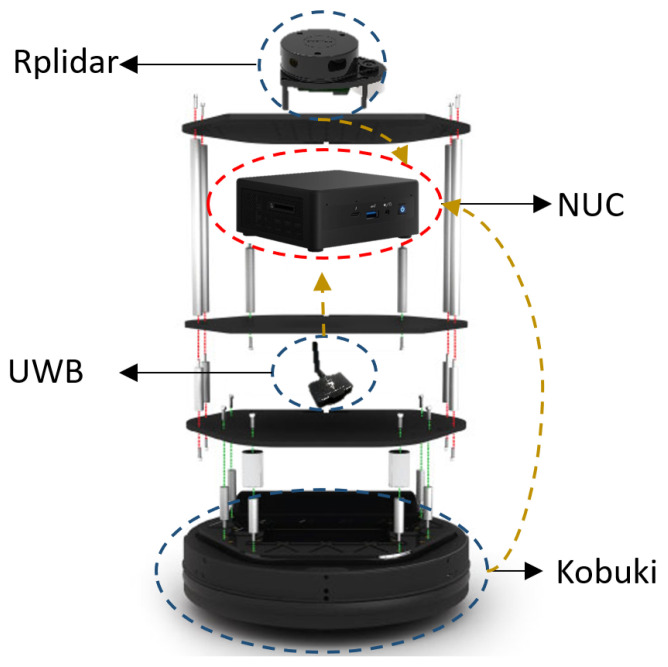
Robot hardware platform.

**Figure 7:**
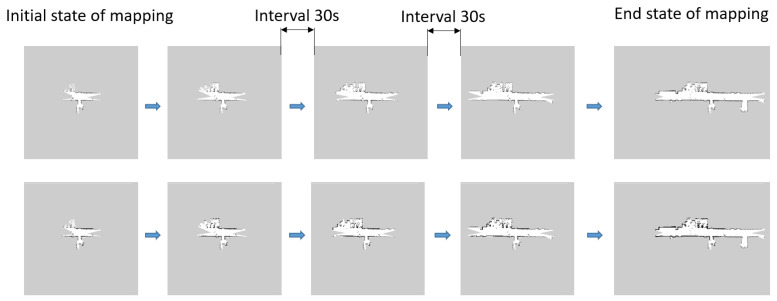
Compressed communication in multi-robot SLAM.

**Figure 8:**
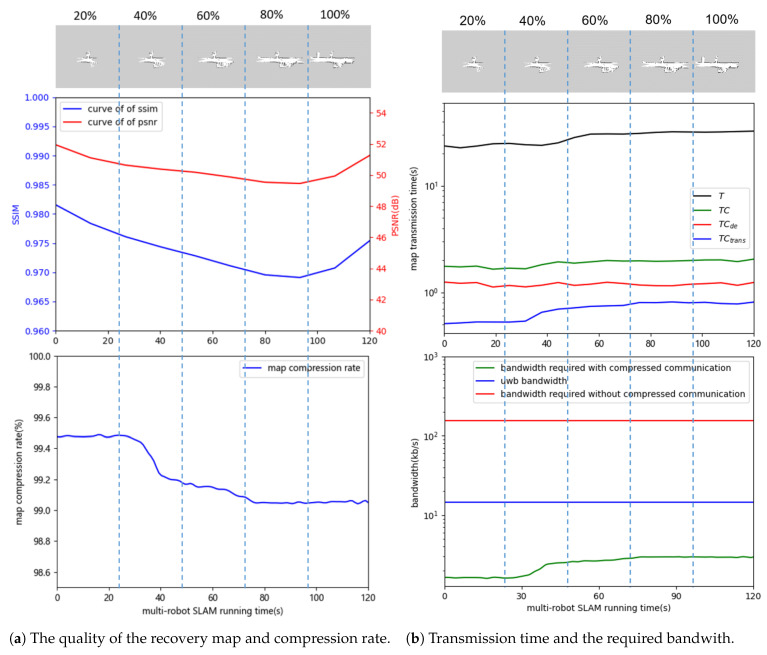
Performance of the proposed methodologies; (**a**) chage in map quality (upper) with change in map compression rate (lower) during the operation of multi-robot SLAM based on compressed communication; (**b**) comparison of map transfer time required with and without compressed communication (upper) versus bandwidth required with and without compressed communication (lower). *T* and TC denote the time to transmit the map using UWB with compressed communication and without compressed communication, respectively, where TC is the sum of $TC_{trans}$ (time to transmit the compressed map) and $TC_{de}$ (time to decompress the compressed map).

In this section, we build the robot platform in Figure 6 and deploy the trained network model to the robots. The performance of the trained model was verified in Figure 7 and Figure 8a. Figure 7 shows the multi-robot SLAM process with and without the compressed communication method, demonstrating that compressed communication has little effect on map quality. Then, multi-robot co-operative SLAM experiments based on the method in this paper are conducted to validate the method further.

### 3.1. Datasets and Model Training

We used the Gmapping algorithm to build maps of different environments. A total of 2,800 occupancy grid maps were saved and used for model training. Model training based on the Pytorch framework was implemented on a computer with AMD Ryzen 5 2600X Six-Core Processor 3.60 GHz (AMD, Santa Clara, CA, USA), NVIDIA GeForce GTX 1050 Ti (NVIDIA, Santa Clara, CA, USA), and 16 G RAM. It takes about 10 h to train the network proposed in this paper.

### 3.2. Robotic Platform

As shown in Figure 6, the robots for collaborative SLAM are mainly composed of a Kobuki drive chassis, an NUC (a microcomputer), Rplidar, and a UWB module. The Gmapping algorithm utilizes information from the chassis’ odometer and Rplidar for full-map creation; then, the full maps are processed by the model deployed in the NUC to get the string. Finally, the string is transmitted via the UWB module. Similarly, the robot receives the string through the UWB module, and it is processed by NUC to get the recovery map. The recovered map is fused with the locally created map to obtain the global map.

### 3.3. Experimental Result

In order to validate the compressed communication frame in Figure 1, we used two robots to collaboratively build maps of a hallway and obtained the map data in Figure 7. In Figure 7, the whole process of the global maps obtained with and without compressed communication for the two robots is shown. The first row of Figure 7 shows the global map obtained without compressed communication; the second row shows the global map obtained with compressed communication; from left to right, the initial state of mapping to the end state of mapping can be seen, and each state of mapping is separated by 30 s.

We evaluated the method of this paper by calculating the metrics for the correspondence maps in Figure 7. The metrics Peak Signal-to-Noise Ratio (PSNR) and Structural Similarity Index Measure (SSIM) were calculated, and the changes in these two metrics with the running time of multi-robot SLAM are plotted in the upper figure of Figure 8a. PSNR is a metric used to quantify the quality of a map, where higher PSNR values typically indicate less distortion introduced by compression and higher map quality. A PSNR above 40 dB indicates excellent map quality (i.e., very close to the original map). On the other hand, SSIM takes values in the range of [−1, 1], with a value closer to 1 indicating more similarity between two maps and a value closer to −1 suggesting greater dissimilarity. The objective is to measure the structural and content similarity between the recovered and original maps. As global map quality depends not only on pixel-level differences but also on factors such as structure and texture, SSIM provides a more accurate assessment of the recovered map’s quality. As shown in the upper figure of Figure 8a, the mean values of PSNR and SSIM are 50.649 dB and 0.975. Therefore, the difference between maps obtained with and without compressed communication is very small, and the quality of maps obtained with compressed communication can be guaranteed. As shown in the lower figure of Figure 8a, the map compression rate of up to 99% significantly reduces the communication burden. Based on the data in Figure 8a, we can conclude that the compression method in this paper has a high compression rate while maintaining good recovery quality. The recovered map can be well utilized to construct the global map.

The upper figure of Figure 8b illustrates the time consumed with compressed communication and without compressed communication. The blue and red lines represent the time for map transmission and decompression, respectively. The black line depicts the total time for a map transmission without compressed communication, while the green line shows the time for a map transmission with compression. As shown in the lower figure of Figure 8b, the blue line (12.5 kb/s) is the bandwidth that can be stably achieved by the UWB model in this framework, and the red (124.7 kb/s) and green (Average 720.4 bit/s) lines represent the bandwidth required for transmission of the map without and with compression, respectively. By using compressed communication, the bandwidth required to transmit the maps is greatly reduced. From Figure 8b, we can conclude that the compressed communication in this paper can satisfy the UWB hardware bandwidth and allow for the fast transmission of maps.

**Figure 9:**
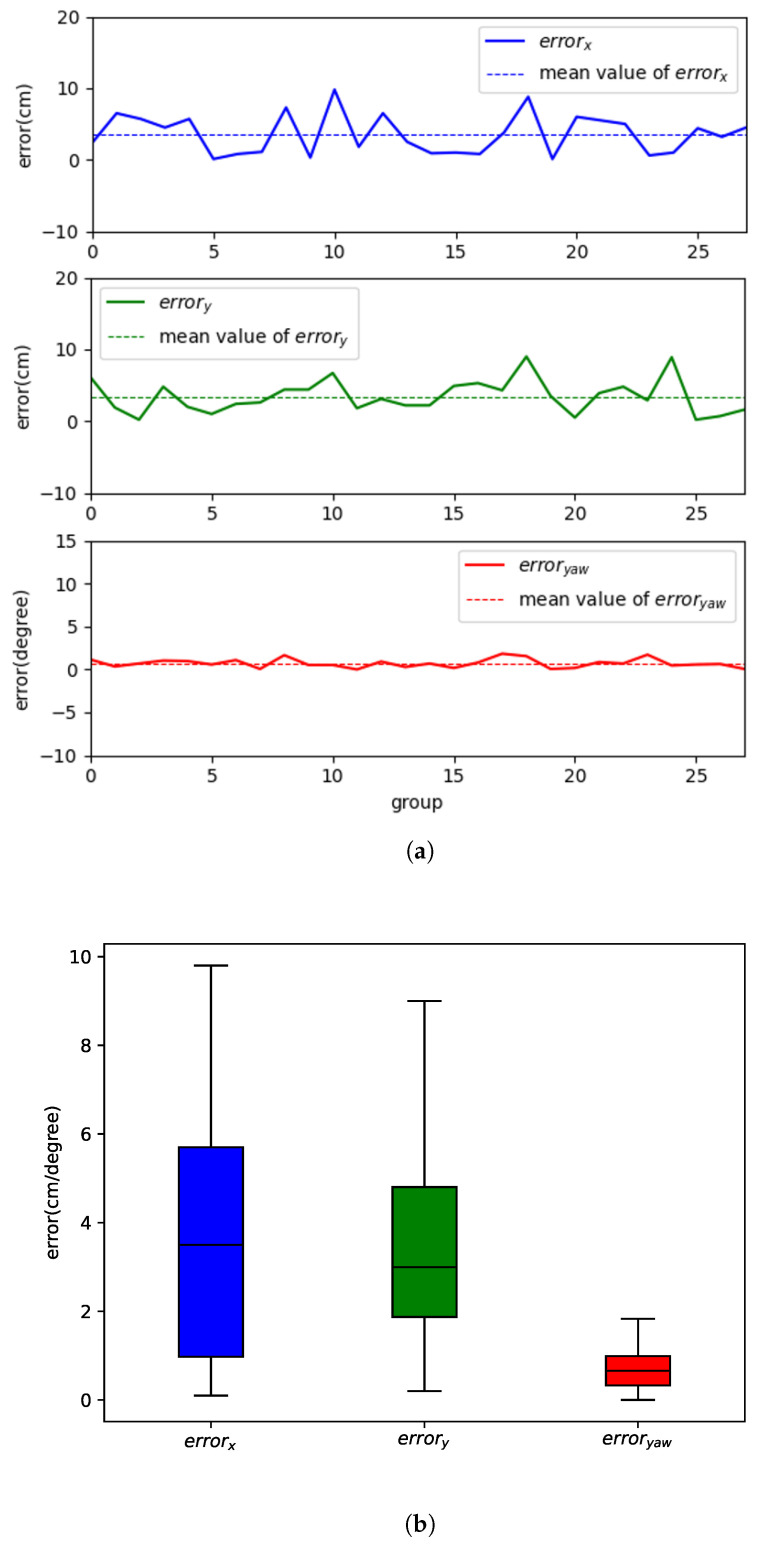
Robot’s localization error using the merged map with compressed communication; (**a**) Performance of localization errors regarding the robot’s position (x,y) and its yaw angle θ; (**b**) Distribution of the localization error over time.

In order to verify the effect of the maps obtained from the compressed communication method in robot localization, we used the occupancy grid map obtained with and without compressed communication to provide localization to the robot, respectively. We selected 28 locations in a realistic environment to cover the entire occupancy grid map. Each location was used separately with compressed and uncompressed maps to provide localization to the robot. We used two sets of data and calculated their errors, as in Figure 9. Figure 9a shows the error variation of the robot in the 28 sets of data, and Figure 9b shows the distribution of the error. The mean values of errors $error_{x}$, $error_{y}$, and $error_{yaw}$ are 3.59 cm, 3.43 cm, and 0.72°, respectively. This result shows that the compressed communication method in this paper can be applied in a multi-robot SLAM and that the subtle changes produced by compressing the map have little effect on robot localization.

## 4. Conclusions

The compressed communication method proposed in this paper consists of the following three main components: (1) full-map creation, (2) full-map compression and transmission, and (3) full-map fusion. We designed a lightweight map-feature extraction CNN and a map recovery CNN that can process occupancy grid maps in real time when processing units with limited arithmetic power. Meanwhile, the encoder and decoder are designed by combining the Huffman and RLE algorithms, which greatly reduce the code length and make the bandwidth required for the transmission code fully satisfy the hardware UWB. The compressed communication framework is validated in a multi-robot system, employing two robots for the collaborative mapping of an unknown environment. One robot compresses and transmits its full map, and the other robot receives and recovers it. Subsequently, the recovery map is fused with its map to generate the global map. The experimental results demonstrate that the method achieves a high compression ratio (99%) and maintains recovered map quality with a PSNR of 50.649 dB and SSIM of 0.975. In summary, the compressed communication method proposed in this paper satisfies the bandwidth-constrained multi-robot SLAM system.

## Figures and Tables

**Table 1:** Comparison of the types of data transferred with the related methods.

Method	Type of Data Transfer	Map Density
Dubé et al. [26]	Segmentation description	Sparse
CVIDS [9]	Descriptor extraction	Sparse
CVI-SLAM [27]	Keyframe and map point	Sparse
$D^{2}$SLAM [28]	Landmarks and descriptors	Sparse
Zhang et al. [24]	Descriptor extraction	Sparse
DCL-SLAM [17]	Descriptor	Sparse
Door-SLAM [15]	NetVlAD descriptor	Sparse
Kimera-Multi [12]	Keypoints and feature descriptors	Dense
Our methoed	2D occupancy full map	Dense

## Data Availability

Data are contained within the article.

## Abbreviations

The following abbreviations are used in this manuscript:

SLAM	Simultaneous Localization and Mapping
CNN	Convolutional Neural Network
RLE	Run-Length Encoding
UWB	Ultra-Wide Band
PSNR	Peak Signal-to-Noise Ratio
SSIM	Structural Similarity Index Measure
